# Antiplatelet Aggregation, Cardiotonic, Anti-Inflammatory, Antioxidant, and Calcium Channel Antagonistic Potentials of *Nepeta ruderalis* Buch

**DOI:** 10.1155/2020/2096947

**Published:** 2020-05-31

**Authors:** Ambreen Aleem, Khalid Hussain Janbaz, Imran Imran, Muqeet Wahid, Sumbal Bibi, Khurram Afzal, Muhammad Hassham Hassan Bin Asad

**Affiliations:** ^1^Faculty of Pharmacy, Bahauddin Zakariya University, Multan, Pakistan; ^2^Institute of Food Science and Nutrition, Bahauddin Zakariya University, Multan, Pakistan; ^3^Department of Pharmacy, COMSATS University Islamabad, Abbottabad Campus 22060, Pakistan; ^4^Institute of Fundamental Medicine and Biology, Department of Genetics, Kazan Federal University, 420008 Kazan, Russia

## Abstract

The objective of this study was to authenticate the ethnobotanical claims of the *Nepeta ruderalis* Buch.-Ham. (*N. ruderalis*) extract in the traditional system of medicine. Crude extract was prepared via a simple maceration process. DPPH free radical scavenging and carrageenan-induced rat paw edema models were used to monitor antioxidant and anti-inflammatory responses of the *N. ruderalis* extract. Furthermore, it was tested for antiplatelet aggregation, cardioprotective, and calcium channel antagonistic activities via standard documented protocols. The *N. ruderalis* extract exhibited 80.82% antioxidant activity (IC_50_ = 207.51 ± 4.36 *μ*g) while the anti-inflammatory response was significant (*p* < 0.05 to *p* < 0.01) at 50 mg/kg (45.58%) and 100 mg/kg (60.90%) doses. Moreover, it was found to inhibit platelet aggregation (IC_50_ = 1.06 and 0.91 mg/mL) and, in addition, to increase the force of contraction at the concentration of 3.0-10 mg/mL with a decrease in the heart rate on isolated paired atria (EC_50_ = 11.78 mg/mL). Relaxant activity was observed on the isolated rabbit jejunum (EC_50_ = 0.96 mg/mL) and trachea (EC_50_ = 0.89 mg/mL). However, in a cumulative way, an 80-millimolar potassium-induced contraction was evaluated (EC_50_ = 1.31 mg/mL). The *N. ruderalis* extract exhibited antioxidant, anti-inflammatory, platelet aggregating, cardiotonic, and calcium channel antagonistic activities, therefore proving scientifically its effectiveness in the traditional system of medicine.

## 1. Introduction

*N. ruderalis* (synonym: *Nepeta hindostana*, Roth; family: Lamiaceae), commonly known as Badranj Boya, is grown wild throughout the Indo-Pak-Bangladesh subcontinent as well as other countries of the Asian continent [1]. It is a medium-sized annual herb with a strong mint-like smell. It bears opposite heart-shaped, green to grayish-green velvety leaves, blue purple flowers, and sturdy stems. It is an annual herb with erect stems of 30-35 cm. The leaves of *N. ruderalis* are broad ovate or triangular ovate. It has an inflorescence of many clearly pedunculated cymes, pedicels up to 3 mm, calyx of 3.5-4 mm, with spreading villous hairs, narrow tubular, throat oblique, and teeth 1/3-1/4 the length of the tube [2, 3].

The various plant parts have been reported to include d-menthone, nepetalic acid, nepetalacton, essential oils, oleanolic acid, nepetanudosides A–D, nepetaside, ajugol, nepetariaside, aucubin, velpetin, nepetin, nepetol, and *β*-sitosterol, nepitrin; 5,9-dehydronepetalactone, and a monoterpene [4, 5]. Other main constituents include a triterpenoid alcohol nepeticin, nepetidin, *β*-sitosterol, glucoside, tetratriacontanol, triterpenic acid, nepehinol, terpenoid nepetidone, nepedinol, nepehinal, and oil rich in sesquiterpene hydrocarbons [6–10]. The plant has traditionally been used as an antiasthmatic, antispasmodic, antidiarrheal, carminative, diaphoretic, sedative, and anti-influenza and to manage multiple cardiovascular ailments including angina pectoris, cardiovascular thrombosis, tachycardia, and congestive heart failure [4, 11]. Moreover, it is known to possess antiaging properties and is used to restore the vigor of old persons. Nepeta species are a famous traditional herbal medicine used as an antioxidant and for anti-inflammation [12]. Scientific studies on the plant reported antiatherosclerotic activity, antifungal activity against Aspergillus and Penicillium species, and cardioprotective, antiprotozoal, antibacterial, and antioxidant activities [10, 13].

Interestingly, *Nepeta ruderalis* Buch.-Ham is claimed to address various disorders by traditional therapists of Pakistan, but there is a lack scientific data for the ethnobotanical uniqueness of this plant. As a result, the ethnobotanical importance of this plant encouraged us to evaluate the scientific basis for its traditional practice in various disorders.

## 2. Material and Methods

### 2.1. Extraction Process

*N. ruderalis* Ham. (aerial parts) was gathered from the hills of Murree, Pakistan, which was recognized by a senior taxonomist from the Department of Pure and Applied Biology of Bahauddin Zakariya University, Multan, and specimen no. “R.R. Stewart F.W. Pak 622(2)” was submitted to the same department. After removal of adulterated material and vegetative debris, plant parts were dried under a shed at room temperature (24 ± 3°C). After shed drying, dried material was grinded into coarse powder via an herbal grinder. The coarse powder of *Nepeta ruderalis* (about 1.0 kg) was triply macerated in 80% ethanol solution in an amber bottle [14]. Firstly, macerated powder filtered through the muslin cloth, subsequently via Whatmann filter paper #1 and evaporated at an optimum temperature (37 ± 3°C) under reduced pressure, to get brownish green residues of the *N. ruderalis* extract (approximate yield of 7.5%) stored at -4°C.

For the experimental purpose, the fresh stock solution of crude extract (0.3 g/mL) in distilled water was prepared with subsequent dilutions on the experiment day. The prepared dilutions of 30 mg/mL, 3 mg/mL, and 0.3 mg/mL of hydroethanolic extract were used in *in vitro* studies using isolated tissues. These dilutions were used to attain isolated tissue bath concentrations of 0.01, 0.03, 0.1, 0.3, 1.0, 3.0, 5.0, and 10 mg/mL.

### 2.2. Standard Drugs and Chemicals

Highly pure analytical grade chemicals, drugs, solvents, and reagents were used in the experiments. Acetylcholine chloride, arachidonic acid, verapamil hydrochloride, calcium chloride, magnesium chloride, carbachol (carbamylcholine), isoprenaline, potassium chloride, adenosine diphosphate (ADP), magnesium sulphate, ethylene tetra-acetic acid, sodium hydroxide, and sodium citrate were procured from Sigma-Aldrich, USA. However, the rest of the chemicals utilized were ordered from Merck (KGaA, Germany) unless and otherwise specified. Fresh stock solution of standard drug was prepared with subsequent dilutions on the experimental day.

### 2.3. Animals and Housing Conditions

The animals, albino rats (weight: 250 to 300 g) and rabbits (weight: 1.0 to 2.0 kg), of either sex, were kept under a controlled environmental condition (i.e., 12 h light and dark rotation, 24 ± 3°C room temperature, and 56 ± 5% humidity) in an animal house situated at B. Z. University. The animals were fed prescribed standard food and *ad libitum* water. All experiments were performed by following the standard guidelines documented earlier in the literature [15]. The approval of animal use has been taken by the committee of ethics to use animals (EC/10/2013).

### 2.4. Antioxidant Activity

The antioxidant activity of the *N. ruderalis* extract was done by DPPH radical scavenging test using propyl gallate as the standard drug with little modifications [16]. The test sample and propyl gallate were allowed to react with 300 *μ*M DPPH (free radical reagent) for 80-90 min at 37°C. The reaction was completed in a 96-well microtiter plate containing 200 *μ*L solution (10 *μ*L of trial/reference sample and 190 *μ*L DPPH) after being vigorously shaken; the mixtures were allowed to incubate for 30 min at 37°C. After incubation, the absorption was measured by a multiplate Elisa reader at 515 nm. The test was performed in triplicate, and the readings were averaged. Percent radical scavenger activity (RSA) was calculated by the given formula:

(1) $$\begin{array}{c} \text{Percentage RSA}=100-\left(\frac{\text{OD test sample}}{\text{OD control}}\times 100\right). \end{array}$$

Afterwards, IC_50_ was calculated by making the five concentrations of the sample beginning with the same concentration and reducing them by twofolds.

### 2.5. Anti-Inflammatory Activity

The *N. ruderalis* extract was tested by the carrageenan-induced rat paw's edema model to scientifically prove its potential to reduce inflammation [17]. Before the experiment, the rats were fasted overnight with free access of water. For experimentation, 20 Swiss albino rats were alienated into four equal groups: group I (control) receives normal saline and group II (standard) receives aspirin (10 mg/kg). Groups III and IV (test drug groups) receive the *N. ruderalis* extract (50 and 100 mg/kg, respectively). Freshly prepared 0.1 mL carrageenan in normal saline was injected 1 h after treatment into the plantar aponeurosis region of the hind paw. At 0, 1, 2, and 3 h of injection, the volume of paw edema was measured by a plethysmometer. The increase of paw volume was used as a parameter for the measurement of inflammation [18].

### 2.6. Antiplatelet Aggregating Activity

The *N. ruderalis* extract was evaluated for antiplatelet activity using ADP and arachidonic acid (inducer of platelet aggregation) as described earlier [19, 20]. In cuvettes, 220 *μ*L aliquots of platelet-rich plasma (PRP) were added and the volume adjusted up to 230 *μ*L with a test sample solution prepared in normal saline or reported vehicle. PRP was obtained from the blood sample of a healthy volunteer after centrifugation at 1000 rpm for 15 minutes at 37°C.

The *N. ruderalis* extract (10 *μ*L) was incubated for 1 minute at various concentrations before challenge with arachidonic acid and ADP (potential platelet aggregation agonists) for 4 min. Hence, platelet exposure to the test sample was approximately 5 min. A lumi-aggregometer (dual channel, Model No 400, Chrono-Log Corporation, USA) measured the antiplatelet aggregating activity, and IC_50_ values of inhibitors were calculated from dose response curves (DRC).

### 2.7. In Vitro Experiments

#### 2.7.1. Cardioprotective Activity

The rabbit paired atria was dissected out and mounted in a tissue organ bath comprising Krebs physiological solution aired with carbogen at 36-37°C after removal of fatty tissues from the atria. The spontaneous beating of isolated atrial preparation was exhibited under 1.0 g tension due to intact pacemaker cells and was permissible to equilibrate for 30 min. The *N. ruderalis* extract was evaluated on an isolated paired atrial preparation for the possible effects on both atrial contractions, i.e., rate and force, and isoprenaline (1 *μ*M) was used as the control inotropic agent. The atrial force of contraction was represented by the amplitude whereas the rate of contraction was represented by the number of contractions; these responses of atrial preparation were recorded through a PowerLab equipped with force displacement transducers having built-in Chart Pro Software (Version 7) [21, 22].

#### 2.7.2. Isolated Rabbit Jejunum Preparation

The *N. ruderalis* extract was exposed for possible spasmolytic activity to jejunum tissue preparations. The jejunum tissue was dissected out from healthy rabbits; after removal of surrounding mesenteries, jejunum preparation (about 2.0 cm) was mounted in a tissue organ bath possessing Tyrode's physiological solution aired with carbogen at 37°C.

Preload tension of about 1.0 g was applied, and responses were recorded through a PowerLab equipped with isotonic transducers having built-in Chart Pro Software (Version 7). Before the addition of drug, the jejunum preparations were allowed to equilibrate for 30 to 40 min and exhibited spontaneous rhythmic contractions. To quantify the observed response of the test sample, the response was recorded immediately before proceeding with a concentration in a cumulative fashion and calculated as the percentage change in spontaneous contractions [23, 24].

For a possible spasmolytic mechanism through calcium channels, the *N. ruderalis* extract was exposed to relax the sustained spasmodic contractions of 80-millimolar potassium in the tissue organ bath; this Ca^2+^ antagonized activity was further confirmed by constructing calcium concentration response curves (CRC) against the preincubated *N. ruderalis* extract as described previously [21, 22]. After stabilization of the jejunum preparations in Tyrode's solution, calcium from tissues is removed by substituting the normal Tyrode's solution with calcium-free Tyrode's solution containing EDTA (0.1 mM) instead of allowing calcium chloride to stabilize in it for 30-40 min. After it, a K^+^-rich Tyrode solution replaced the tissue organ bath. After a 30 min incubation period, Ca^2+^ concentrations were added in a cumulative manner to construct superimposable control calcium concentration response curves (CRCs) usually after 2-3 cycles. The tissues were washed away and incubated with the *N. ruderalis* extract for 50-60 min, and CRCs were constructed against the *N. ruderalis* extract compared to the respective controls.

#### 2.7.3. Isolated Rabbit Trachea Preparation

*N. ruderalis* extract was exposed to rabbit tracheal preparations for possible bronchodilator effects. The trachea was dissected out from healthy rabbits, after removal of surrounding fatty substances from the trachea, and was segmented into a wide ring preparation (approximately 3-4 mm); these rings were cut in such a manner that the smooth muscle was sandwiched between the cartilage portions of the trachea. The tracheal preparation was suspended in a tissue organ bath containing Krebs physiological solution aired with carbogen at 37°C.

A preload of 2-3 g tension was applied, and isometric responses were recorded through a PowerLab equipped with force displacement transducers having built-in Chart Pro Software (Version 7). Before the addition of any drug, the tracheal preparation was allowed to equilibrate for 60 ± 10 min. For a possible bronchodilator response of the *N. ruderalis* extract on a precontracted tracheal preparation with 80-millimolar potassium and carbachol to quantify the observed response of the test sample, the response was recorded immediately before proceeding with a concentration in a cumulative fashion and calculated as percentage change contractions.

### 2.8. Statistical Analysis

The data was reported as mean ± SEM and EC_50_ via GraphPad Prism v7 software. Anti-inflammatory activity was analyzed via two-way ANOVA while Dunnett's test was found significant at *p* < 0.05.

## 3. Results

The *N. ruderalis* extract showed 80.82% activity when tested for DPPH radical scavenging activity, while propyl gallate showed 92.29% activity. The median effective concentration (IC_50_) of the *N. ruderalis* extract was estimated to be 207.51 ± 4.36 *μ*g (Tables 1 and 2), while the linear regression showed that the IC_50_ of *N. ruderalis* was found to be 420 *μ*g as compared to propyl gallate with an IC_50_ value of 143 *μ*g.

**Figure 1:**
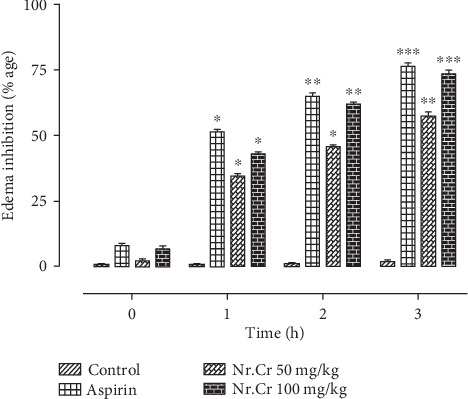
Effects of aspirin and *N. ruderalis* extract on rat paw volume as compared to control. Values shown are mean ± SEM (*n* = 5). Data was evaluated by ANOVA (two-way) when compared to control, and *p* < 0.05 was considered significant.

*N. ruderalis* extract exhibited anti- inflammatory activity when tested by a classical *in vivo* model. *N. ruderalis* extract was found significantly (p <0.05 to p <0.01) active at doses 50 mg/kg and 100 mg/kg and showed 60% and 73.33% inhibition, respectively at the 4^th^ h of inflammation (Table 3, Figure 1).

**Figure 2:**
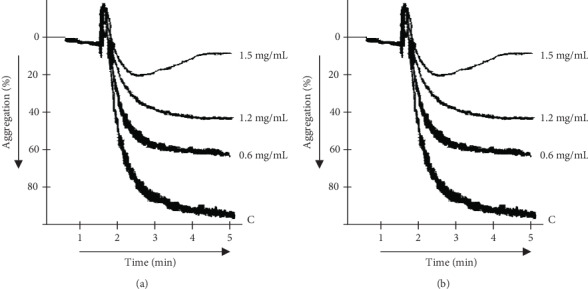
Tracing showing the inhibitory response of different concentrations of crude extract of *N. ruderalis* on (a) ADP-induced aggregation in humans and (b) arachidonic acid-induced aggregation in human platelets.

Aspirin (10 mg/kg) presented 76% inhibition and was found significantly active contrary to the control. The *N. ruderalis* extract inhibited the platelet aggregation in a concentrated fashion (0.3 to 1.2 mg/mL) induced by ADP and arachidonic acid, and the median inhibitory concentration (IC_50_) was assessed to be 1.06 mg/mL and 0.91 mg/mL (Figures 2(a) and 2(b)), respectively.

**Figure 3:**
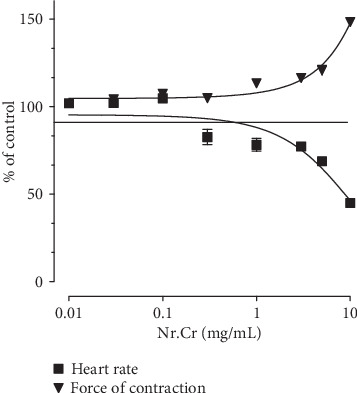
Effect of hydroethanolic extract of *N. ruderalis* extract on heart rate and force of contraction in guinea pig atria. The data show mean ± SEM (*n* = 5).

The *N. ruderalis* extract exhibited positive inotropic with negative chronotropic effect, i.e., increase in force of contraction of myocardium, while decease in heart rate on isolated atrial preparations in a cumulative manner within the concentration range of 3.0-10 mg/mL and EC_50_ 11.78 mg/mL (95% CI: 2.89-47.97; *n* = 5) (Figure 3).

**Figure 4:**
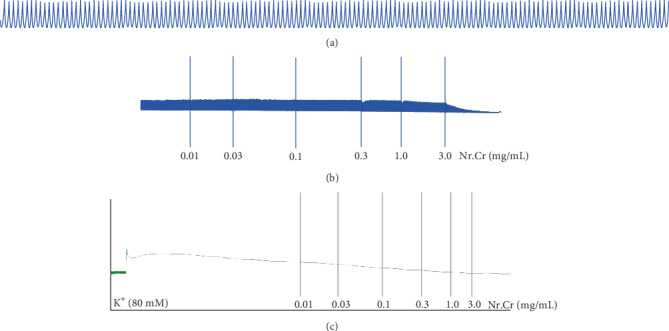
Tracing showing (a) the spontaneous contracting isolated jejunum (control) and (b) the effect of hydroethanolic extract of *N. ruderalis* extract on spontaneous contractions on (c) 80-millimolar potassium-induced contractions in isolated rabbit jejunum preparations.

**Figure 5:**
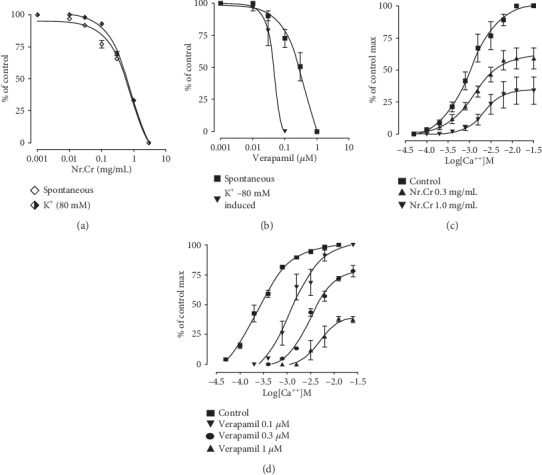
Inhibitory effect of (a) hydroethanolic extract of *N. ruderalis* extract and (b) verapamil on spontaneous periodic and 80-millimolar potassium-induced contractions in jejunum preparation. The calcium concentration-response curves of the (c) crude extract of *N. ruderalis* extract and (d) verapamil. The data show mean ± SEM (*n* = 5).

The cardiotonic effect was resistant to propranolol, when the experiment was repeated in propranolol (3 *μ*M) pretreated tissues. When spontaneous periodic contractions of isolated jejunum preparations were treated with the *N. ruderalis* extract in a cumulative fashion in the tissue organ bath, it exhibited the relaxant effect within a concentration range of 0.1-3.0 mg/mL with EC_50_ of 0.96 mg/mL (95% CI: 0.72-1.27; *n* = 5) (Figures 4(a), 4(b), and 5(a)). Moreover, *N. ruderalis* extract instigated the relaxant activity in a cumulative fashion on 80-millimolar potassium-induced contractions within the concentration range of 1-3.0 mg/mL with EC_50_ of 1.31 mg/mL (95% CI: 0.96-1.76; *n* = 5) (Figures 4(c) and 5(a)); this spasmolytic effect was comparable to verapamil (standard Ca^2+^ channel antagonist) which exhibited the relaxant activity on spontaneous periodic contractions and 80-millimolar potassium-induced contractions with EC_50_ value of 0.384 *μ*M (95% CI: 0.27-0.53) and 0.057 *μ*M (95% CI: 0.03-0.09, *n* = 5), respectively (Figure 5(b)). Additionally, concentration response curves (CRC) of calcium were constructed, to confirm the Ca^2+^ ion channel antagonist activity of the *N. ruderalis* extract; the isolated jejunum preparation pretreated with the *N. ruderalis* extract at various concentrations (0.3 to 0.1 mg/mL) markedly suppresses the CRC of calcium and shifted the curves to the rightward direction similar to verapamil (Figures 5(c) and 5(d)).

**Figure 6:**
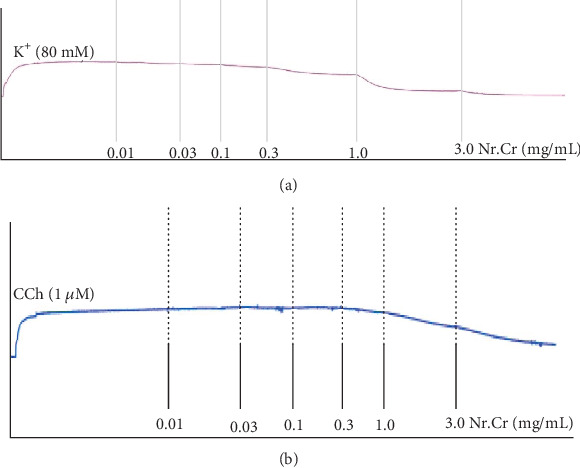
Concentration-dependent inhibitory effect of hydroethanolic extract of *N. ruderalis* extract on (a) 80-millimolar potassium- and (b) carbachol- (1 *μ*M) induced contractions in isolated rabbit tracheal preparations.

**Figure 7:**
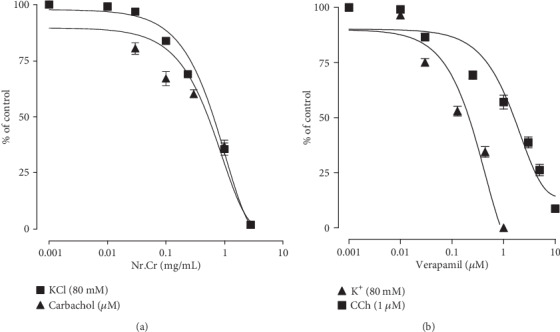
Inhibitory effect of (a) hydroethanolic extract of *N. ruderalis* extract and (b) verapamil on 80-millimolar potassium- and carbachol- (1 *μ*M) induced contractions in rabbit tracheal preparation. The data show mean ± SEM (*n* = 5).

To authenticate the folkloric use of the *N. ruderalis* extract, it was evaluated for possible bronchodilator activity. Crude extract instigated the relaxant activity in a cumulative fashion on 80-millimolar potassium- and carbachol- (1 *μ*M) induced contractions within the concentration range of 3.0 mg/mL with EC_50_ of 0.89 mg/mL (95% CI: 0.50-1.66; *n* = 5) and 1.34 mg/mL (95% CI: 1.04-1.73; *n* = 5) (Figures 6 and 7(a)), respectively. Comparison with the abovementioned EC_50_ values reflects that EC_50_ of the *N. ruderalis* extract for K^+^- (80 mM) induced contractions was numerically less than that of CCh- (1 *μ*M) induced contractions in isolated tracheal preparations, which is likely to be viewed that the *N. ruderalis* extract may exert a relaxant effect through the blockade of the Ca^2+^ channels comparable to verapamil, which caused relaxation of 80-millimolar potassium- and CCh-induced contractions with EC_50_ values of 0.087 *μ*M (95% CI: 0.05-0.13; *n* = 5) and 0.09 *μ*M (95% CI: 0.04-0.09; *n* = 5), respectively (Figure 7(b)).

## 4. Discussion

The crude hydroethanolic extract of *N. ruderalis* showed significant antioxidant, anti-inflammatory, antiplatelet aggregating, cardioprotective, and calcium channel blocking activities. Antioxidants act as essential factors in health protection and help to reduce the risk of life-threatening chronic diseases like tumours (benign or malignant) and heart diseases. DPPH is commonly used as free radical scavengers and/or hydrogen donors as well as to evaluate antioxidant activity [25]. The change in color of the reaction mixture from deep purple to yellow and the decrease in absorbance at the wavelength of 517 nm indicated the scavenging of the DPPH radical. The *N. ruderalis* extract was tested for its antioxidant potential and found to be 85.5% effective in radical scavenging and can be effective as an antioxidant agent and help in preventing various diseases.

The calcium accumulation in cells causes noxious stimuli which induce inflammation and nociception by releasing some mediators such as bradykinin, cytokines, histamine, prostaglandin, serotonin, and substance P [26, 27]. The verapamil and nifedipine (standard calcium channel blockers (CBC)) antagonized inflammation induced by carrageenan had been previously reported, which indicated the role of Ca^2+^ influx in inflammation [28]. In the carrageenan-induced rat's paw edema model, the *N. ruderalis* extract showed significant anti-inflammatory activity at 50.0 mg/kg (*p* < 0.05) and 100.0 mg/kg (*p* < 0.01). The acute inflammation is reported to be biphasic due to the release of inflammatory mediators: (1) in the early phase (1-2 h), histamine and serotonin were released and produced edema in paws and (2) the late phase (after 2 h) releases bradykinins, cytokines, prostaglandins, and substance P which mediated the vascular permeability [29]. The crude extract exhibited a strong anti-inflammatory effect in the third hour compared to aspirin, which might be through the cyclooxygenase enzyme inhibition in the arachidonic acid pathway or which might be because of the presence of calcium channel blocking activity in the *N. ruderalis* extract [30, 31]. As described earlier, cyclooxygenase inhibition also suppresses the platelet aggregation; hence, anti-inflammatory and antiplatelet aggregation activities are linked together [32]. The *N. ruderalis* extract with an anti-inflammatory nature exhibited an antiplatelet aggregation effect and significantly blockaded ADP and arachidonic acid-induced platelet aggregation.

The *N. ruderalis* extract has folkloric repute for use in cardiovascular disorders, i.e., angina pectoris, cardiac thrombosis, tachycardia, and heart failure. The *N. ruderalis* extract was tested on isolated preparations of rabbit atria to explore its possible effect on inotropic and chronotropic activities. The *N. ruderalis* extract exhibited a positive inotropic effect and a negative chronotropic effect. Some previous studies suggested that calcium channel blockers like felodipine, nifedipine, verapamil, and (+)-*cis*-diltiazem showed a positive inotropic response in isolated perfused hearts. Referring to these studies, the observed positive inotropic effect owed to an indirect effect through increased systolic ventricular pressure produced by vasodilatation. Coronary vasodilatation instigated by calcium channel blockers increased the fibre tension of the myocardium and, according to the Frank-Starling mechanism, increased the myocardial contractile strength [33]. The observed negative chronotropic effect may likely be due to the proposed nonselective calcium channel blocking effect (CCBs) present in the extract.

The calcium antagonistic activity was further studied on isolated jejunum and trachea tissue preparations of rabbit to explore its folkloric uses. The *N. ruderalis* extract exhibited a relaxant effect (0.1-3.0 mg/mL) on spontaneous periodic contractions of isolated jejunum. These contractions occur due to episodic depolarization followed by repolarization of the cell via voltage-dependent L-type calcium channels (VDLCs); the depolarization action potential was produced due to the fast influx of the Ca^2+^ current [21, 22]. The repolarization or suppression of periodic contractions was caused either by a blockade of Ca^2+^ channels and antagonism receptors or by the open activity of K^+^ channels [34]. Contraction of smooth muscles depends on intracellular Ca^2+^ concentration; elevated Ca^2+^ concentration levels bind with an acidic nature protein known as calmodulin; this Ca^2+^-calmodulin complex causes the phosphorylation of myosin by activating myosin light chain kinases (MLC kinase) [35].

To confirm the Ca^2+^ channel blockade effect of the *N. ruderalis* extract on isolated tissue preparations, the tissue was exposed to 80-millimolar potassium which depolarized the cell and induced sustained contractions through the opening of VDLCs, and materials able to relax contractions induced by 80-millimolar potassium are presumed to be Ca^2+^ influx blockers [23, 36, 37]. The *N. ruderalis* extract revealed the relaxant effect on contractions induced by 80-millimolar potassium in both isolated tissue preparations, i.e., the jejunum and trachea preparations, in a manner compared to verapamil. These speculations were confirmed by constructing concentration response curves (CRCs) against preincubated the *N. ruderalis* extract on isolated tissue preparations. The *N. ruderalis* extract decreased the CRCs and caused rightward-shift CRCs, in a manner comparable to verapamil [35]. Calcium channel blockers are well reputed due to their therapeutic application in hyperactive smooth muscle disorders like diarrhea and asthma [38, 39].

## 5. Conclusion

The crude hydroethanolic extract of *N. ruderalis* revealed the presence of antioxidant, anti-inflammatory, antiplatelet aggregating, cardiotonic, spasmolytic, and bronchodilator activities by *in vivo* and *in vitro* experiments. The observed relaxant effect in cardiac and smooth muscles may be a consequence of Ca^2+^ channel blockade activity. The obtained results offer a basis for the folkloric use of this plant in managing inflammation and disorders related to the cardiovascular, gastrointestinal, and respiratory systems and are another step to provide evidence for practice of phytomedicine.

## Figures and Tables

**Table 1:** DPPH radical scavenging activity of hydroethanolic extract of *N. ruderalis* extract at various concentrations.

Extract	Concentrations (*μ*g)	% RSA	IC_50_ ± SEM (*μ*g)
*N. ruderalis* extract	1000	85.52	207.51 ± 4.36
	500	58.63	
	250	33.61	
	125	24.64	
	62.5	22.97	

**Table 2:** DPPH radical scavenging activity of propyl gallate at various concentrations to compare the effect of *N. ruderalis* extract.

Compound	Concentrations (*μ*g)	% RSA	IC_50_ ± SEM (*μ*g)
Propyl gallate (PG)	500	90.83	30.00 ± 2.00
	250	83.79	
	125	75.92	
	62.5	40.71	
	31.25	36.42	

**Table 3:** Percentage inhibition of hydroethanolic extract of *N. ruderalis* extract at various time intervals on carrageenan-induced rat paw edema model.

Treatments	Dose	% edema inhibition			
		0 h	1 h	2 h	3 h
Control	—	—	—	—	—
Aspirin	10 mg/kg	8.3	51.02	64.7	76
*N. ruderalis* extract	100 mg/kg	0	42.85	60.29	73.33

Note: data was evaluated by ANOVA (two-way) when compared to control, and *p* < 0.05 was considered significant.

## Data Availability

Upon request, data may be provided by Ambreen Aleem.
